# Pathogenesis of Inflammation in Skin Disease: From Molecular Mechanisms to Pathology

**DOI:** 10.3390/ijms251810152

**Published:** 2024-09-21

**Authors:** Simona N. Shirley, Abigail E. Watson, Nabiha Yusuf

**Affiliations:** 1 Heersink School of Medicine, University of Alabama-Birmingham, Birmingham, AL 35233, USA; sns2022@uab.edu; 2 College of Medicine, Florida State University, Tallahassee, FL 32306, USA; aew20c@med.fsu.edu; 3 Department of Dermatology, University of Alabama-Birmingham, Birmingham, AL 35294, USA

**Keywords:** neuro-immune crosstalk, TLRs, NLRP inflammasome, JAK/STAT, AHR/ARNT, non-coding RNA, psoriasis, atopic dermatitis, hidradenitis suppurativa, scleroderma

## Abstract

Many skin diseases begin with inflammatory changes on a molecular level. To develop a more thorough understanding of skin pathology and to identify new targets for therapeutic advancements, molecular mechanisms of inflammation in the context of skin disease should be studied. Current research efforts to better understand skin disease have focused on examining the role of molecular processes at several stages of the inflammatory response such as the dysregulation of innate immunity sensors, disruption of both transcriptional and post-transcriptional regulation, and crosstalk between immune and neuronal processes (neuro-immune crosstalk). This review seeks to summarize recent developments in our understanding of inflammatory processes in skin disease and to highlight opportunities for therapeutic advancements. With a focus on publications within the past 5 years (2019–2024), the databases PubMed and EBSCOhost were used to search for peer-reviewed papers regarding inflammatory molecular mechanisms and skin disease. Several themes of research interest regarding inflammatory processes in skin disease were determined through extensive review and were included based on their relative representation in current research and their focus on therapeutic potential. Several skin diseases such as psoriasis, atopic dermatitis, hidradenitis suppurativa, and scleroderma were described in the paper to demonstrate the widespread influence of inflammation in skin disease.

## 1. Introduction

Deep within the core of all anatomic and clinical pathology, cellular components and their complex interactions form the basis of disease. Thus, the secret code to understanding and managing skin disease lies in the land of submicroscopic changes and the miniscule, yet majorly important mechanisms of the body’s molecules. Molecular pathology, or the study of disease through the examination of intracellular and extracellular constituents such as proteins and nucleic acids, underpins much of current knowledge regarding the causes of skin disease and the effective targets for therapeutic strategies. Every day, the scientific community’s appreciation for molecular sources of skin disease expands as new studies emerge. This literature review, which examines the inflammatory mechanisms of skin disease at a molecular level, seeks to highlight current areas of interest in the field and exciting new advancements in the community’s understanding of dermatopathology. After introducing each relevant component of pathogenic inflammation and identifying the specific mechanisms most widely addressed in the recent literature, the details of each process as they apply to individual skin diseases will be discussed. Whether they are classified as autoimmune, autoinflammatory, or both autoimmune and autoinflammatory, all disease processes included in the review involve pathogenic inflammatory mechanisms in the skin. Importantly, all included diseases also represent potential candidates for the emerging avenues of anti-inflammatory therapies mentioned in the review.

### 1.1. Innate Immunity Sensors

The first step in launching an inflammatory immune response is the recognition of a noxious insult such as infection, tissue injury, or tissue stress by sensors of the innate immunity system [1]. One important group of such sensors are the pattern recognition receptors (PRRs). PRRs not only respond to triggers of the immune system, but they also mediate the initial response of immune cells such as recruiting neutrophils to the site of inflammation [1]. The triggers PRRs respond to are molecular patterns from microbes or pathogens (MAMPs or PAMPs) and cell damage or death-associated molecular patterns (DAMPs) [2]. While PPRs can be categorized into four different groups of receptors—RIG-like receptors (RLRs), NOD-like receptors (NLRs), C-type lectin receptors (CLRs), and Toll-like receptors (TLRs)—the two types of PRRs which have been conserved from early invertebrates to mammals and which represent the current area of focus in dermatological research studies are NLRs and TLRs [1,3].

**Figure 1:**
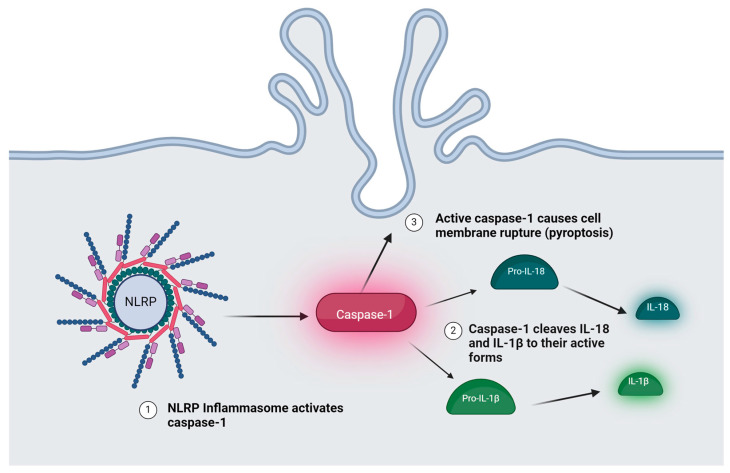
Inflammasome Response. Created with BioRender.com. (Accessed on 10 July 2024).

NLRs are found in the cell cytosol, and they are multi-domain-containing proteins. The domains include a C-terminal domain with leucine-rich repeats (LRRs), a central nucleotide-binding NACHT domain, and an N-terminal effector domain [3]. Classification of NLRs is based on the variable N-terminal domain. The five families of NLRs are the NLRAs, NLRBs, NLRCs, and the NLRPs [3]. The NLRP subfamily has been identified as particularly relevant to inflammation pathogenesis in dermatological disease processes, especially because of its role in forming the inflammasome. The NLRP1 and NLRP3 members of the NLRP subfamily are particularly important for inflammasome formation. Inflammasomes are cytoplasmic protein complexes which contribute to inflammatory processes by indirectly activating cytokines IL-1β and IL-18 [4]. Inflammasomes directly activate caspase-1 proteins which then proteolytically cleave cytokines IL-1β and IL-18, releasing their active forms. Active IL-1β and IL-18 are responsible for inducing inflammation [4]. Active caspase-1 can also lead to cell pyroptosis by causing cell membrane rupture. Each type of inflammasome contains an NLRP in its center [4]. The *NLRP1* variant is considered the predominant inflammasome in human keratinocytes [5]. While the NLRP1 inflammasome was the first inflammasome studied, the NLRP3 inflammasome is the most well studied [5]. Differential expression and genetic polymorphisms of NLRP1 and NLRP3 have been associated with several skin diseases [3]. Inhibitors of the NLRP inflammasomes, such as the NLRP3-specific inhibitor MCC950 and the ADS032 inflammasome inhibitor, which binds to both NLRP1 and NLRP3, are being studied as potential therapeutic agents for certain skin diseases [5] [Figure 1].

**Figure 2:**
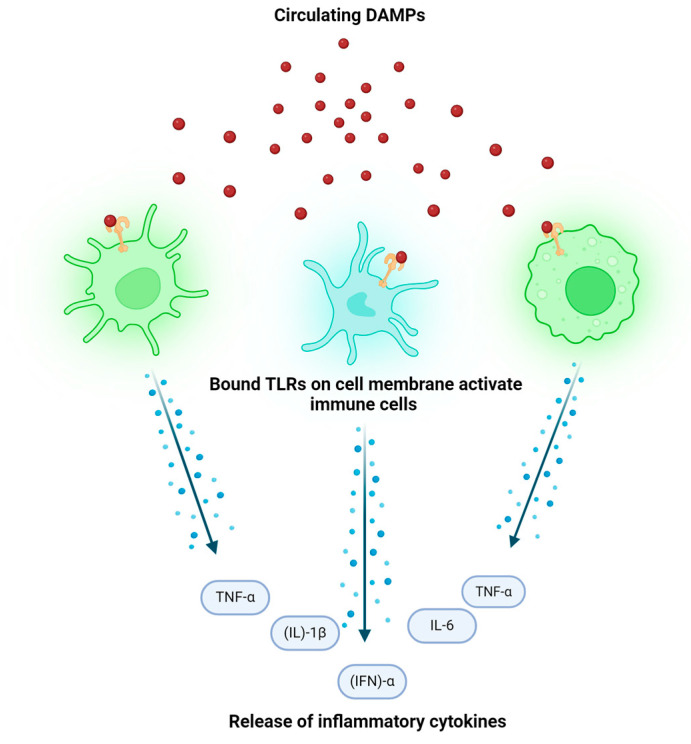
TLR Response. Created with BioRender.com. (Accessed on 10 July 2024).

Unlike NLRs, which reside in the cytosol, TLRs are transmembrane receptors [6]. When bound, they trigger the activation of pro-inflammatory signal transduction pathways which release several different inflammatory cytokines such as tumor necrosis factor (TNF-α) and interferon (IFN)-α [6]. The innate immune cells involved in inflammatory processes of the skin such as Langerhans cells, dermal dendritic cells, macrophages, mast cells, and innate lymphoid cells all become activated and release inflammatory cytokines once the different TLRs in their cell membranes are bound by innate immunity triggers such as DAMPs [6] [Figure 2].

### 1.2. Transcriptional Regulation

**Figure 3:**
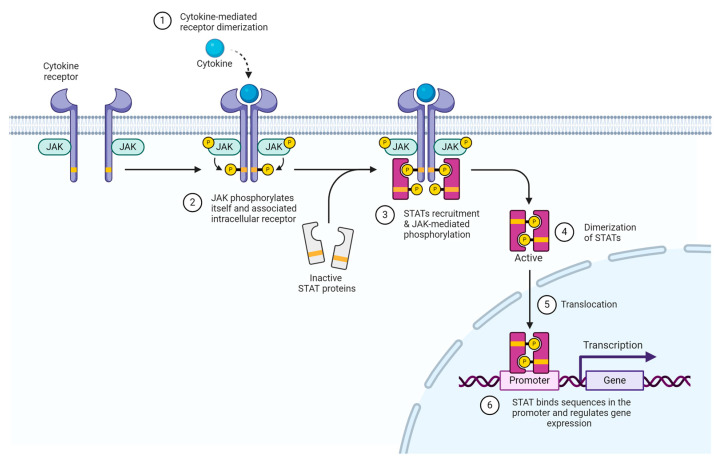
JAK/STAT Pathway. Adapted from“Cytokine Signaling throughe JAK-STAT Pathway” by BioRender.com. (Accessed on 10 July 2024).

Once innate immunity sensors respond to the molecular patterns that indicate cell stress of pathogen invasion, intracellular signaling involves a pathway of interconnected ligands and enzymes which transmit and amplify messages inside the cell. Many times, the purpose of such signaling is to reach the cell nucleus and influence gene expression by interacting with transcription factors. By targeting transcription factors and triggering the ultimate production of more inflammatory proteins, inflammation can become more widespread and persistent. One particularly important molecular process involved in regulating gene transcription in the context of skin disease and the target of widely used pharmacological therapies is the Janus Kinase/Signal Transducers and activators of the transcription (JAK/STAT) pathway [7]. The JAK/STAT pathway is well known for its simple and direct communication from transmembrane receptors to transcription factors in the nucleus. Janus kinases (JAKs) become activated once their associated transmembrane receptor is bound by a cytokine ligand [7]. Once they are activated, they phosphorylate themselves and the intracellular component of their associated receptor. STAT (signal transducer and activator of transcription) protein from the cytoplasm moves to the membrane and binds JAK and its receptor [7]. JAK proceeds to phosphorylate STAT, which in turn becomes activated, dimerizes, and travels to the nucleus as an active transcription factor for regulating gene expression [7]. JAK inhibitors (JAKi) such as tofacitinib, ruxolitinib, and baricitinib have already been used for therapeutic management of several skin diseases classified as either inflammatory or autoimmune [8]. Due to their selective blockade of certain cytokine groups, they are considered a valuable alternative to traditionally used immunosuppressants such as cyclosporine and corticosteroids, which have more widespread effects and less specific targets [8] [Figure 3].

**Figure 4:**
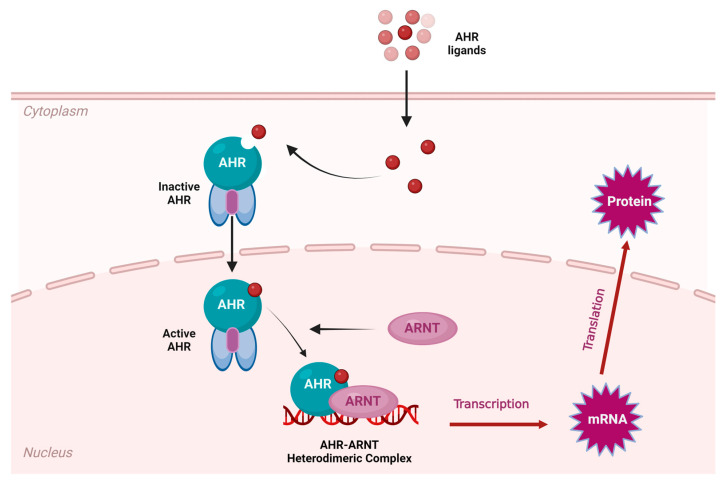
AHR-ARNT Pathway. Adapted from“AHR signaling pathway” by BioRender.com (2024). (Accessed on 10 July 2024).

Recent investigations concerning the molecular pathology of skin disease have also revealed the significance of the intracellular receptor AHR (aryl hydrocarbon receptor). AHR can be activated by both exogenous and endogenous ligands such as photo-induced chromophores, phytochemicals, and microbial byproducts [9]. AHR also functions as a transcription factor [9]. After being activated by a ligand, AHR signaling may undergo two major pathways: the canonical and non-canonical pathways. In the canonical pathway, also known as the AHR-ARNT signaling pathway, AHR undergoes a conformational change upon ligand activation which allows it to translocate from the cytoplasm to the nucleus [10]. ARNT (aryl hydrocarbon receptor nuclear transporter) facilitates AHR’s migration to the nucleus. Once the ligand-bound AHR reaches the nucleus, it forms a heterodimeric complex with ARNT and becomes a high-affinity DNA-binding transcription factor [10]. This complex then binds to specific DNA sequences to regulate gene transcription. The identification of a non-canonical pathway, in which AHR does not translocate to the nucleus but instead remains in the cytoplasm or plasma membrane and interacts with other signaling cascades, adds further complexity to the emerging role of AHR in the molecular pathology of skin disease [10]. AHR signaling, and its subsequent influence on the expression of genes related to the immune response, is known to play a significant role in the pathogenesis of skin inflammation [10] [Figure 4].

### 1.3. Post-Transcriptional Regulation 

Beyond the transcriptional level, expression of inflammation-related genes can also be modulated through post-transcriptional epigenetic changes which affect the translation process and, ultimately, the amount and types of proteins that are produced. Many post-transcriptional modifications rely on the actions of non-coding RNAs (ncRNAs) [11]. NcRNAs are not translated into proteins themselves, but they can regulate the translation of other proteins [11]. NcRNAs can be classified as small ncRNAs and long ncRNAs. Long ncRNAs are more than 200 nucleotides long, and short ncRNAs are less than 200 nucleotides [11].

One specific type of short ncRNA that has been identified as a potential biomarker and therapeutic target for several skin diseases is microRNA (miRNA). MiRNAs are only about 21–25 nucleotides in length [12]. MiRNA regulates gene expression post-transcriptionally by degrading certain parts of mRNA sequences or by binding mRNA and inhibiting its translation [11,12]. Differences in miRNA expression affect how various immune cells such as B and T lymphocytes, macrophages, and dendritic cells are expressed; therefore, inflammatory processes may change according to the state of the cells’ miRNA. Several different inflammatory diseases such as rheumatoid arthritis and inflammatory bowel disease exhibit both upregulation and downregulation of specific miRNA types; now, similar differences in miRNA expression are being identified for inflammatory skin diseases such as atopic dermatitis and psoriasis [11]. Several studies have identified miRNA as an influential factor in the pathogenesis of autoimmune and inflammatory skin diseases [11].

Long non-coding RNAs (lncRNAs) are a large and diverse group of ncRNAs; tens of thousands of different lncRNAs exist in the human genome [12]. Like the short ncRNA miRNA, lncRNAs have also been implicated in the pathogenesis of skin inflammation. LncRNAs support the development of skin disease by causing differential expression of certain types of mRNA and protein. LncRNAs may be particularly useful as biomarkers of disease because they exhibit highly specific expression patterns in different cells and tissues [12].

**Figure 5:**
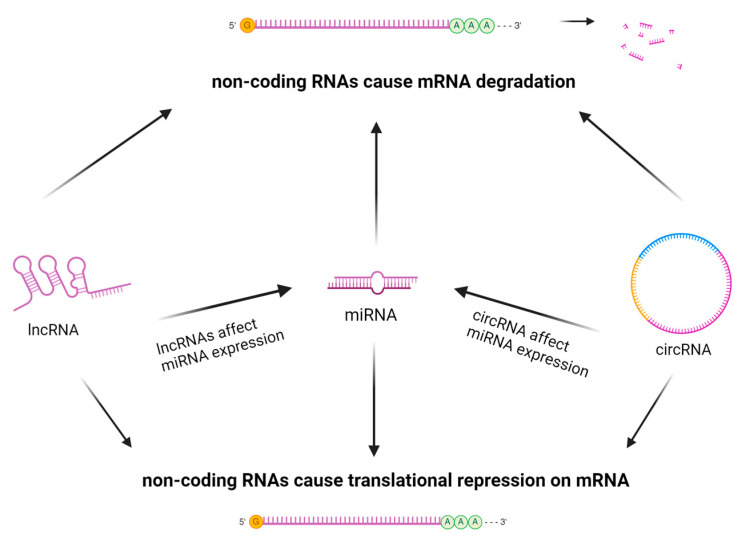
Post-Transcriptional Regulation of Non-coding RNAs. Created with BioRender.com. (Accessed on 10 July 2024).

Another type of ncRNA which is not classified as either sncRNA or lncRNA is circular RNA (circRNA). The uniquely circular structure of circRNA, which is held together by covalent bonds, protects it from exonuclease attack [13]. CircRNA has been implicated in the regulation of several skin processes such as wound healing, keratinocyte differentiation, and melanin production [13]. CircRNA exhibits differential expression in the context of several inflammatory disease processes of the skin, and it acts as an miRNA sponge, meaning it can bind to miRNA and therefore block miRNA’s interaction with target mRNA [13]. CircRNAs, miRNAs, and lncRNAs may all function as valuable assay markers because they are easily detectable in serum, urine, and saliva samples [12] [Figure 5].

### 1.4. Neuro-Immune Axis

**Figure 6:**
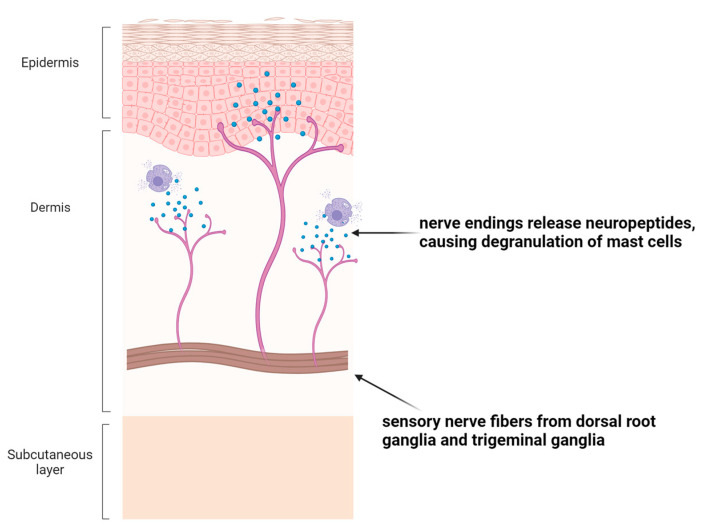
Example of Neuro-Immune Interaction in Skin. Created with BioRender.com. (Accessed on 10 July 2024).

Recent scientific developments have revealed how inflammatory processes not only rely on changes within the traditional immune system such as the activation of immune cells and inflammatory cytokines, but they are also mediated by interactions between the immune system and the nervous system [14]. Sensory nerve fibers, which are responsible for transmitting sensations such as pain and itch, are located in close proximity to active immune cells in the epidermis and dermis of the skin [14]. All the sensory nerve fibers which innervate the skin carry excitatory signals, and they originate from the dorsal root ganglia (DRG) and trigeminal ganglia (TG) [15]. Components of both the nervous system and immune system are thought to work together to amplify inflammation through a mechanism known as neuro-immune crosstalk. Neuro-immune crosstalk is critical for maintaining homeostasis in the skin, but it can also contribute to the pathogenesis of inflammation in skin disease [14]. For example, certain neuropeptides and neurotransmitters can trigger the degranulation of mast cells from the immune system, which ultimately results in pruritus—a significant symptom of several skin diseases [14] [Figure 6].

## 2. Psoriasis 

Psoriasis is an inflammatory skin disease experienced by about 2% of the worldwide population [16]. Compared to Asian and African American populations, it shows a higher prevalence in Caucasian and Scandinavian populations [16]. The most common type of psoriasis is psoriasis vulgaris, or plaque-type psoriasis, with other types of psoriasis including pustular psoriasis, inverse psoriasis, and guttate psoriasis. It presents clinically as sharply demarcated erythematous plaques with scales, commonly appearing on the extensor surfaces of limbs, the scalp, and the trunk; however, more severe cases may cover the skin more extensively as the plaques may coalesce [17]. Additionally, psoriasis of the nails affects more than half of psoriasis patients [16]. Psoriasis is associated with both autoimmune and genetic causes. It is considered a systemic disease because the inflammation affects other systems in the body beyond the skin. For example, psoriasis is associated with metabolic syndrome, cardiovascular disease, arthritis, gastrointestinal involvement such as inflammatory bowel disease, chronic kidney disease, and even depression and anxiety [16].

Pathogenic proliferation of epidermal keratinocytes due to chronic inflammation characterizes the molecular pathology of psoriasis. In general, psoriasis pathogenesis occurs in two stages: the initiation phase and the maintenance phase. The initiation phase begins with a stress factor that triggers disease such as trauma, infection, or drugs. Plasmacytoid dendritic cells (pDCs) respond to the initiating stressor. The initial response is thought to involve the stimulation of toll-like receptors (TLRs) on pDCs by certain peptides released by injured keratinocytes called antimicrobial peptides (AMPs) [18]. Once activated, pDCs produce type 1 interferons (IFN-α and IFN-β), which then trigger the development of myeloid dendritic cells (mDCs) [16]. Myeloid dendritic cells secrete cytokines TNF-α, IL-12, and IL-23 to stimulate the differentiation and proliferation of the Th17 and Th1 subtypes of T cells in the adaptive immune response. During the maintenance phase, cytokines released from T helper cells act to increase the number of epidermal keratinocytes and to trigger inflammation within the cell through intracellular signaling pathways [16,19]. The Th1 cytokine IFN-γ in particular was found to trigger keratinocyte proliferation in vitro [19]. Nevertheless, targeting Th1 cytokines such as IFN-γ and TNF-α alone was not shown to improve psoriasis outcomes [19]. In contrast, targeting the Th17 cytokines IL-17 and IL-23 did result in therapeutic benefits for psoriasis patients [19]. In addition to Th1 and Th17, Th22 cells are also thought to play a role in psoriasis pathogenesis. Th22 cells were found to be increased in the skin of psoriasis patients, and a deficiency in the Th22 cytokine IL-22 was found to decrease skin thickening and inflammation [19]. Although additional inflammatory pathways contribute to psoriasis pathogenesis, especially in the context of less common psoriasis subtypes such as inverse or guttate psoriasis, the described Th1/Th17/Th22-mediated immune response is a well-defined and prominent mechanism in the pathology of psoriasis vulgaris, the most common type of psoriasis. It also represents the target of several pharmacological treatments for the disease.

### 2.1. Innate Immunity Sensors (TLRs and NLRP Inflammasomes) in Psoriasis 

TLRs are crucial for initiating the immune response in psoriasis. Keratinocytes and pDCs respond to DAMPs and PAMPs by binding their TLRs and subsequently producing proinflammatory cytokines such as IFN-β and TNF-α [20].The initial release of proinflammatory cytokines promotes the primary T-cell-mediated response of inflammation in psoriasis [20]. Certain polymorphisms regarding TLR expression may play a role in establishing the severity of disease in psoriasis patients and also their responsiveness to certain treatments. The TT genotype polymorphism of TLR9, a TLR found on pDCs, was strongly associated with a high (>10) Psoriasis Area Severity Index (PASI), and the TT genotype also showed decreased responsiveness to standard narrow-band ultraviolet B light therapy when compared to TC and CC genotype polymorphisms of TLR9 [2,20]. Clinical trials have demonstrated the emerging benefits of using TLR9 antagonists for suppressing inflammatory pathways in psoriasis pathogenesis. The TLR9 antagonists IMO-3100 and IMO-8400 were shown to block activation of the IL-17 pathway in moderate to severe plaque psoriasis [20]. Several mouse model studies of psoriasis use imiquimod, a TLR7 and TLR8 agonist, to induce the formation of psoriasiform skin plaques on the mice [2]. Not all TLRs are shown to promote the formation of psoriasis, however. A mouse model study assessing the function of TLR2s in psoriasis pathogenesis surprisingly found that a deficiency in TLR2s actually caused more psoriasis-like skin inflammation and downregulated the protective anti-inflammatory agents IL-10 and regulatory T cells (Tregs) [21]. More studies are needed to determine the different roles of *TLR* variants in the pathogenesis of inflammation in psoriasis. 

Regarding the NLRP inflammasome, certain single-nucleotide polymorphisms (SNPs) of the NLRP1 inflammasome gene have been associated with a risk of developing psoriasis. Such mutations cause excessive stimulation of the inflammasome, therefore increasing the amount of inflammatory cytokines in the cell [22]. One example of an implicated SNP is the *rs878329C* allele; homozygosity for the *rs878329C* allele significantly increases risk of early-onset psoriasis [23]. Differences involving the NLRP3 inflammasome have also been associated with psoriasis. Skin samples from psoriasis patients showed that the NLRP3 inflammasome was expressed at a level four times higher than in normal skin [23]. NLRP3 polymorphisms have also been associated with psoriasis susceptibility [23]. Further study of *NLRP* variations in psoriasis patients, as well as their downstream effects on inflammation, may reveal the therapeutic role of inhibiting NLRP inflammasomes for psoriasis treatment.

### 2.2. Transcriptional Regulation (JAK/STAT and AHR Pathways) in Psoriasis

The JAK-STAT intracellular signaling pathway has been highly implicated in psoriasis pathogenesis. JAK-STAT inhibitors have been approved by the USFDA for psoriasis treatment. The Th22 inflammatory cytokine IL-22, a major player in psoriasis pathology, activates JAK proteins TYK2 and JAK1 [24]. Another cytokine IL-23, which is released by Th17 cells in the psoriasis immune response, activates JAK2 and tyrosine kinase 2 (TYK2) [24]. Interestingly, individuals with a genetic polymorphism which causes loss of function of TYK2 are at lower risk of developing psoriasis [8]. While the helper T cell Th17 seems to lead the course of inflammation in psoriasis pathology, Th1 helper cells also contribute. The Th1 inflammatory cytokine IFN-γ activates two JAK proteins: JAK1 and JAK2 [24]. Several JAK inhibitor drugs (JAKi) have been approved for psoriasis treatment, and many more are currently under investigation [8]. Topical formulations of JAKi have proven particularly useful because, instead of dispersing systemically and potentially causing systemic adverse effects, they tend to accumulate in the epidermis and dermis where psoriasis pathogenesis occurs [8].

Another molecular mechanism linked to inflammatory processes in psoriasis is dysregulation of the AHR pathway. Certain AHR mutations which lead to overstimulation of the receptor seem to correlate with psoriasis susceptibility. Overactive AHRs may bind to ligands such as tryptophan metabolites inside the cell and pollutants from the external environment, leading to increased transcription of inflammatory cytokines and the subsequent process of epidermal hyperplasia [10]. While AHR stimulation may increase the inflammatory response, and AHR antagonists are currently under investigation as a therapeutic option for psoriasis, the AHR pathway also plays a regulatory role in preventing the overexpression of inflammatory cytokines [9]. In a seemingly counterintuitive manner, AHR deficiency leads to increased inflammation as well because inflammation mechanisms are left unregulated. In a study of AHR-deficient mouse models, the mice demonstrated increased IL-22 and IL-17 cytokines, as well as psoriasis-like skin inflammation [10]. Moreover, the recently approved drug Tapinarof acts as an AHR agonist and regulates the expression of Th17 cells; Tapinarof has shown success as a topical treatment for psoriasis [9]. Reaching a balance between under expression and overstimulation of the AHR pathway appears to be key for the management of psoriasis.

### 2.3. Post-Transcriptional Regulation (miRNA, lncRNA, circRNA)in Psoriasis

Many dysregulated miRNAs have been identified in psoriasis pathogenesis. They work together to promote the dysregulation of epidermal keratinocytes. Some, such as miR-210 and miR-318, also function in disrupting the balance of T helper cells [25]. They selectively increase the expression of the psoriasis-involved T cells, Th17 and Th1, and inhibit the protective mechanisms of T regulatory cells [25]. Using the well-established imiquimod-induced mouse model, it was discovered that miR-214-3p is significantly underexpressed in the skin of psoriasis-like lesions. MiR-214-3p downregulation contributes to psoriasis pathology by causing increased expression of the transcription factor FOXM1; FOXM1 increases cell proliferation by controlling cell cycle dynamics [26]. Cell proliferation leads to the characteristic epidermal hyperplasia of psoriasis. It was also shown how correcting the miRNA deficiency through the administration of miR-214-3p could, in turn, decrease FOXM1 expression and slow the hyperproliferation of keratinocytes [26]. Several miRNA subtypes may also function as biomarkers of disease. Tracking miRNA levels may aid in diagnosing psoriasis earlier and in assessing the success of treatment strategies. One clinical study of psoriasis patients found that increased expression of miRNA-223 correlated with more severe disease [27]. The study stressed how tracking miRNA-223 levels could be used as a marker of disease severity and an early warning sign for progression of disease [27].

CircRNAs have also been implicated in the pathogenesis of psoriasis by acting as sponges to certain miRNAs and therefore inhibiting their actions. For example, the upregulation of has_circ_0061012 is associated with psoriasis pathogenesis because it targets miR-194-5p, an miRNA which usually inhibits the migration, proliferation, and invasion of keratinocytes [13]. In contrast, downregulation of circRAB3B is associated with psoriasis pathogenesis because circRAB3B usually sponges an miRNA known as miR-1228-3p which is known to inhibit the pathogenic processes of psoriasis [13]. Several other types of circRNA have been studied in the context of psoriasis pathogenesis. Another example is hsa_circ_0003738, which was found in the dysfunctional Treg cells of psoriatic skin [28]. It was found that targeting hsa_circ_0003738 and causing its downregulation could restore the immunosuppressive function of Treg cells in psoriasis [28]. Some studies have suggested that circRNA expression could be used for psoriasis screening and diagnosis [28].

Finally, lncRNAs also seem to influence the pathogenesis of inflammation in psoriasis. More than 2194 lncRNAs were dysregulated in psoriatic lesions according to microarray analysis [28]. The lncRNAs are thought to contribute to keratinocyte dysfunction and inflammation. For example, one lncRNA called PRINS (psoriasis susceptibility-related RNA gene induced by stress) was found to be associated with susceptibility to psoriasis [28]. Interestingly, PRINS was found to maintain keratinocyte hyperproliferation, but it decreased the expression of IL-6 and IL-8, two inflammatory cytokines involved in the inflammatory processes of psoriasis [28]. The lncRNA maternally expressed gene 3 (MEG3) appears to induce autophagy and inflammation in psoriasis [29]. Blocking the expression of MEG3 reserved TNF-α-mediated autophagy and inflammation [29]. Other lncRNAs such as antisense noncoding RNA in the INK4 locus (ANRIL) and Kelch domain containing 7B (KLHDC7B)-DT induce the activation of IL-6 and IL-8 and therefore promote inflammation [28]. Targeting specific lncRNAs in therapeutic strategies for psoriasis may prove beneficial by reducing inflammation.

### 2.4. Neuro-Immune Axis in Psoriasis

Interaction between components of the nervous system and the immune system are critical for the pathogenesis of inflammation in psoriasis. Several neuropeptides such as substance P (SP), calcitonin gene-related peptide (CGRP), and vasoactive intestinal polypeptide (VIP) were shown to be upregulated in psoriatic skin [14]. Increased interaction between mast cells and neural components was also seen in psoriatic skin [14]. A higher density of nerve fibers and mast cells in skin correlates with greater disease severity in psoriasis [30]. Interactions between the neuropeptides and mast cells induces inflammation by causing mast cells to release inflammatory cytokines IL-1β and TNF-α, and it contributes to the complex pathogenesis of itch in psoriasis [14]. While mast cells classically contribute to pruritus by releasing histamine, histamine release does not seem to play a significant role in the pathogenesis of psoriasis. Instead, neuropeptides are thought to alter the function of mast cells in psoriatic skin by promoting the expression of cathepsin B, a pro-inflammatory protease [31]. Cathepsin B is upregulated in the mast cells of psoriatic skin, and it allows for the activation of tryptase, an enzyme released by mast cells to induce psoriatic pruritus [31]. Targeting *CTSB*, the gene responsible for cathepsin B, may present the opportunity for a novel anti-inflammatory therapy for psoriasis [31]. 

Dermal dendritic cells, which are critical for initiating the inflammatory cascade in psoriasis, are also influenced by neural interactions. Communication between transient receptor potential V1+ cells and dermal dendritic cells (DCs) triggers DCs to produce IL-23, a prominent cytokine in psoriasis inflammatory processes [14]. The somatosensory neuron ASH1L was found to decrease expression of miR-let-7b, an miRNA which increases inflammation in psoriatic lesions by acting as a ligand for TLR7s on DCs and causing them to initiate inflammation [30]. Thus, further stimulation of lysine-specific methyltransferase 2H (ASH1L) may prove beneficial for decreasing inflammation in psoriasis.

## 3. Atopic Dermatitis 

Atopic dermatitis (AD), also known as atopic eczema, is an inflammatory skin disease which mostly affects pediatric populations. The frequency of AD among pediatric populations is up to approximately 20% [32]. About 1 in 10 people worldwide will have experienced atopic eczema at least once in their lives [33]. Most AD patients first experience symptoms by age 5 [33]. AD is highly associated with other atopic conditions such as allergic rhinitis and asthma [33]. Its clinical presentation is highly variable and differs with age. In children, AD usually appears as erythematous papules, patches or plaques. However, AD tends to present more as patches rather than papules or plaques as children get older [33]. For younger children, the erythema tends to be located on the cheeks, scalp, trunk, and extremity. The erythematous patches on older children, however, are usually limited to the flexural surfaces [33]. Adults with AD mostly experience dry, scaly patches on the extremities [33]. The most common symptom of AD is pruritus, which is difficult to manage for many patients and is perpetuated by the self-destructive itch–scratch cycle [33]. Patients with AD are also at high risk for infection, particularly by the bacteria *Staphylococcus aureus* [33].

In general, the pathogenic mechanisms of AD all relate to one major problem: disruption of the skin barrier. Structural and functional problems concerning the epidermis, particularly the top layer called the stratum corneum, can leave the skin vulnerable to antigen invasion, pH imbalance, and dryness from water loss [32]. Downregulation of the protein filaggrin is one of the most well-defined causes of AD [32]. Filaggrin plays an integral role in building the protective envelope around keratinocytes in the epidermis. The specialized keratinocytes in the stratum corneum are called corneocytes, and the protective envelope is referred to as the cornified envelope [32]. Mutations in the *FLG* gene encoding filaggrin can disturb the integrity of the cornified envelope by decreasing filaggrin activity. Genetic mutations may also downregulate other proteins involved in maintaining the skin barrier such as claudin and occludin, which form intercellular tight junctions [32]. Beyond genetic changes, epigenetic influences such as DNA methylation and post-transcriptional modifications by miRNA are also thought to be highly implicated in AD pathogenesis [32]. While genetic mutations are common amongst AD patients, they are not required for the development of AD [32].

Immunological mechanisms also downregulate the expression of filaggrin and other proteins which maintain skin barrier function. Whether the external barrier disruption or the internal immunological changes occur first in the pathogenesis of AD is still unknown. Nevertheless, skin barrier dysregulation and the immune response work hand in hand [32]. Innate lymphoid cells type 2 (ILC2) are thought to initiate the adaptive immune response by activating Th2 lymphocytes [32]. Th2 lymphocytes primarily drive the process of AD inflammation. They secrete cytokines IL-4, IL-5, and IL-13, which stimulate the production of IgE antibodies and eosinophils while decreasing filaggrin expression [32]. Damaged cells from the epidermis release more inflammatory cytokines such as thymic stromal lymphopoietin (TSLP). TSLP also contributes to the proliferative actions of cytokines IL-4, IL-5, and IL-13 [32]. While the Th2 pathway has proven most prevalent, other T helper cells such as Th22, Th17, and Th1, along with their corresponding proinflammatory cytokines, have also been associated with AD inflammation mechanisms [32]. Action by Th1 lymphocytes such as the activation of IL-2, IL-12, TNFα, and INF cytokines particularly corresponds with the chronic phase of AD [32].

### 3.1. Innate Immunity Sensors (TLRs and NLRP Inflammasome) in Atopic Dermatitis

Several TLRs contribute to the early inflammatory processes of AD. TLR2, which usually responds to infectious agents such as *S. aureus* and activates an immune response to remove the pathogen in normal skin, has decreased expression in the AD-affected skin [2]. Therefore, patients with AD are more susceptible to infection by *S. aureus*, a microbial organism which causes more inflammation in AD pathogenesis [2]. Expression levels of TLR3 were found to be elevated in the stratum corneum of AD-affected skin [34]. TLR3 expression levels also correlated with several markers of disease severity such as total intensity score, erythema score, and oozing/crusting score [34]. TLR3 deficiency has been associated with decreased pruritus because TLR3 signaling promotes the expression of nerve growth factor (NGF) and TSLP, two proteins involved in the mechanisms of pruritus in AD [34]. Therefore, targeting TLR3 may help with decreasing scratching behavior in AD patients. Several studies have assessed the prevalence of *TLR1*, *TLR4*, and *TLR1* SNPs in AD patients and have found certain polymorphisms which seem to increase susceptibility to disease [2,34]. Further investigation is needed to solidify association between TLR polymorphisms and risk of developing AD.

Recent attention to inflammatory signaling pathways in AD pathology emphasizes the involvement of NLRP inflammasomes. A recent clinical study evaluated the expression of NLRP1 and NLRP3 inflammasomes, as well as their associated cytokines IL-18 and IL-1β, in AD-affected skin [35]. NLRP1 and NLRP3 were overexpressed in the dermal layer of skin, and levels of IL-18 and IL-1β cytokines were significantly increased in the epidermal layer [35]. The degree of NLRP1 overexpression was also linked to the relative severity of disease in AD patients [35]. Genetic polymorphisms of both the NLRP1 and NLRP3 inflammasomes have been associated with the development of AD; one Swedish study identified the NLRP1 SNP *rs12150220* as a risk factor for AD [36]. Another clinical study supported the idea that NLRP1 inflammasome overactivation may be caused by microbiome disruption in AD [37]. The study showed how areas with higher accumulation of NLRP1 inflammasome were the same areas where particles of the invading bacteria *Staphylococcus aureus* were most abundant. It is thought that *Staphylococcus aureus* proteases may be responsible for NLRP1 activation and the subsequent release of inflammatory cytokines IL-18 and IL-1β [37]. Activation of the NLRP3 inflammasome appears particularly central to the pathogenesis of AD. Several natural compounds from traditional medicine, such as the flavonoid glucoside icariin, *Coffea arabica* extract (CAE), sodium thiosulfate (STS), a homogenous polysaccharide from the herb Lonicera japonica, and another herbal formula called Angelica Yinzi, have all proven to alleviate inflammation in AD by downregulating the NLRP3 inflammasome [38,39,40,41,42].

### 3.2. Transcriptional Regulation (JAK/STAT and AHR Pathways) in Atopic Dermatitis

The relevance of JAK-STAT signaling mechanisms to the pathophysiology of AD is well accepted in the dermatology community. Just as they are for psoriasis, JAK-STAT inhibitors have been approved for AD treatment [24]. The major Th2 inflammatory cytokines of AD such as IL-4, IL-5, IL-13, IL-31, and TSLP all use the JAK-STAT pathway to exert their effects [24]. Therefore, blocking the JAK-STAT pathway may harness significant benefits for preventing AD inflammation. JAK-STAT mechanisms are also involved in the pathways responsible for causing pruritus in AD. Activation of STAT3 in particular was shown to cause activation of astrocytes in the spinal dorsal horn, a mechanism linked to the development of chronic pruritus in AD patients [24]. As JAK-STAT transduces inflammatory signals from cytokines into DNA transcription, it promotes the translation of certain cellular components which contribute to skin barrier disturbance [43]. For example, too much stimulation of JAK1 causes overexpression of cutaneous serine protease, an enzyme responsible for disrupting intercellular connections in the epidermis [43].

Regarding the role of the AHR in AD, evidence suggests that moderate stimulation of AHR may protect the skin barrier by stimulating the release of filaggrin and increasing the rate of keratinocyte differentiation [10]. As previously stated, filaggrin deficiencies are highly implicated in AD pathology. Also, faster differentiation of keratinocytes leads to enhancement of the skin barrier [10]. While the exact mechanisms for how filaggrin production and keratinocyte differentiation improve with AHR stimulation, current evidence suggests how AHR activation may increase expression of Ovo-like-1 (OVOL1), a transcription factor responsible for transcribing the DNA of several important proteins for epidermal integrity such as filaggrin [9]. Certain treatments, such as the historical use of coal tar for AD and the new AD medication Tapinarof, are both thought to activate AHR as their mechanism of action against AD [9]. Topical application of the AHR-agonist Tapinarof has shown significant success in restoring skin barrier function for AD patients [9]. Rather than repressing AHR, allowing for more AHR stimulation may be key to future therapeutic strategies in AD.

### 3.3. Post-Transcriptional Regulation (miRNAs, lncRNAs, circRNAs) in Atopic Dermatitis

Recent investigations of epigenetic influences in AD molecular pathology reveal the significance of differentially expressed miRNAs. Like in psoriasis, many different types of miRNA are either overexpressed or downregulated in AD-affected skin. In fact, psoriasis and AD share 77 differentially expressed miRNAs in common [44]. Changes in miRNA expression influence the development and maintenance of AD through several different mechanisms such as regulating components of the skin barrier, disrupting proteins and cytokines involved in intracellular signaling, and slowing the rate of keratinocyte differentiation. For example, MiR-939 upregulation was found to increase matrix metalloproteinases and cell adhesion protein intracellular adhesion molecule 1 (ICAM1) in AD-affected keratinocytes from a human skin sample [44]. Such differences in the extracellular matrix structure and function promoted colonization by *Staphylococcus aureus*, the main bacteria involved in the characteristic skin infections of AD [45]. The miRNA subtype miR-378a-3p was also shown to induce *Staphylococcus aureus* infection and therefore the progression of AD. MiR-378a-3p was upregulated in AD-affected skin; its upregulation affected the expression of IL-33, a cytokine responsible for AD’s inflammatory response to *Staphylococcus aureus* infection [45].

Other miRNAs connected to AD pathogenesis work on different aspects of inflammation. While overexpression of miR-146a inhibits parts of the NF-κB pathway, miR-155 upregulation promotes the concentration of the proinflammatory cytokines IL-2, INF-γ, and IL-17 [11]. Additional miRNAs that are upregulated in AD-affected skin such as miR-10-5p disrupt the skin barrier by slowing the differentiation and proliferation of keratinocytes. MiR-10-5p directly inhibits the damage-associated positive regulator hyaluronan synthase 3 (HAS3), which usually promotes the maturation of keratinocytes [46]. While inhibiting some of the overexpressed miRNAs involved in AD pathology may prove useful for future advancements in AD treatment, administering additional miRNA may also cause therapeutic benefits. For example, miR-143 has been shown to protect the integrity of the skin barrier by blocking the actions of IL-13, a cytokine which disrupts the normal function of vital skin barrier proteins such as filaggrin, loricrin, and involucrin [47]. Therefore, supplying additional miRNA such as miR-143 to AD-affected skin may protect the skin barrier and provide therapeutic effects for patients with AD as well.

While current knowledge regarding the mechanisms of lncRNA in AD pathogenesis remains sparse and more investigation is needed, one mouse model study found that downregulating the lncRNA metastasis associated lung adenocarcinoma transcript 1 (MALAT1) by applying an herbal extract called Morina officinalis extract (MOE) caused decreased inflammation and improved AD symptoms in the mice [48]. Previous studies have indicated that, of the more than 1000 lncRNAs expressed in the skin, around 40% may be dysregulated in AD lesional skin [49]. The lncRNAs differentiation antagonizing non-protein coding RNA (DANCR), terminal differentiation-induced ncRNA (TINCR), and H19, which all play a role in maintaining epidermal homeostasis, were found to be downregulated in AD lesional skin [49]. Other important lncRNAs such as urothelial cancer associated 1 (UCA1), which promotes the NFκB (Nuclear factor kappa-light-chain-enhancer of activated B cells) inflammatory pathway, were upregulated in AD lesional skin [49].

The role of circRNAs in the inflammatory processes of AD should also be studied more extensively. So far, a circRNA microarray revealed that the circRNA hsa_circ_004287 is upregulated in the peripheral blood mononuclear cells of AD [50]. The circRNA hsa_circ_0004287 was found to alleviate AD inflammation by inhibiting M1 macrophages [50]. Therefore, further activating of hsa_circ_0004287 could be a potential therapeutic strategy for AD. While many similarities exist between the expression of different circRNAs in AD and psoriasis lesional skin, one study identified ciRS-7 as a specific circRNA which was uniquely elevated in AD while much less abundant in psoriasis [51]. The study suggested the potential use of ciRS-7 as a biomarker for AD [51].

### 3.4. Neuro-Immune Axis in Atopic Dermatitis

Recent studies have highlighted the significance of neuro-immune mechanisms in the pathogenesis of AD, particularly focusing on the roles of neuropeptides and their interactions with various immune cells. Neuropeptides can influence inflammation, vascular dynamics, and sensory perceptions such as pain and itch, which are hallmark symptoms of AD [52]. In AD, an increased density of nerve fibers has been observed in the epidermis, dermal papilla, and around blood vessels in the skin lesions of patients [52]. This hyperinnervation suggests that nerve fibers and the neuropeptides they release may significantly contribute to the pathophysiology of AD. Neuropeptide Y (NPY) is a crucial neuropeptide with a widespread distribution in the body, including sympathetic and sensory nerve endings [53]. Besides its expression in these locations, NPY is also produced by various immune cells such as lymphocytes, monocytes, and chromaffin cells [53]. NPY has been shown to influence the function of mast cells, Langerhans cells, and monocytes, which are integral to the immune response in AD. Research has demonstrated that NPY plays a role in modulating itch signaling pathways in the spinal cord, particularly in inhibiting mechanical itch [53]. While its primary function appears to be the inhibition of mechanical itch, there is evidence to suggest that NPY may also suppress histamine-induced itch and IL-31-mediated itch, both of which are prominent in AD.

Substance P (SP) is another neuropeptide that has been implicated in the neuro-immune interactions underlying AD. Elevated levels of SP have been detected in AD patients, along with an increased number of SP-expressing nerve fibers, monocytes, and epidermal cells expressing neurokinin 1 receptor (NK1-R) [54]. SP can be released from various cells, including nerve fibers, mast cells, monocytes, keratinocytes, and eosinophils. Upon release, SP activates receptors such as MrgGPRX2, MrgGPRB2, and NK1-R on mast cells, leading to their degranulation [53]. This degranulation process results in the production of pro-inflammatory mediators like TNF-α, leukotriene B4 (LTB4), prostaglandins, and histamine, which contribute to vasodilation, plasma extravasation, and the sensation of itching [54].

## 4. Hidradenitis Suppurativa 

Hidradenitis suppurativa (HS) usually begins after puberty, and it most commonly presents within the second and third decade of life [55]. It is thought to affect between 1 and 4% of the population [56]. While the initial clinical presentation varies, patients usually present with erythematous subcutaneous nodules, accompanied by pruritus and discomfort in areas of the skin such as the axillary, inguinal, anogenital, and inframammary regions [56]. The skin locations most affected by HS share a common feature: they all bear a high density of apocrine sweat glands. In fact, inflammation or infection of the apocrine glands was initially hypothesized to be the primary mechanism of HS. However, further investigation has revealed how HS really begins with follicular occlusion [56]. Eventually, occluded hair follicles dilate and rupture, releasing their contents into the surrounding area and triggering an inflammatory response. The chronic inflammation from repeated occlusion and rupture eventually causes tunnel formation in the dermis skin layer and the development of deep-set abscesses and skin ulceration [56]. The skin’s attempts to heal results in fibrosis and disfigurement of the affected skin. HS causes significant physical and psychological suffering. Besides the associated pain and itching, the malodorous purulent discharge from ruptured follicles and the extensive scarring cause social embarrassment and isolation for many patients [55]. While patients’ needs are far from met with current treatment strategies, research efforts in recent years have revealed significant insight into the molecular pathology of HS, pointing toward a more hopeful future.

As previously stated, local inflammation drives the pathogenesis of HS. While bacterial infections may occur secondarily, they are not the primary cause of disease [56]. High levels of the inflammatory cytokines TNF-α and IL-1β have been identified in HS lesions [57]. Like in psoriasis, the IL-1β/IL-23/Th17 axis is thought to be the primary pathway of HS inflammation. Increased levels of IL-23 cytokines activate the T helper cells from the Th17 family, which then secrete IL-17 cytokine, an activator of innate immunity mechanisms such as neutrophil recruitment [57]. The T helper cell Th1, as well as its mediators, also appear to play a supporting role in HS inflammation. However, HS-affected skin is deficient in Th22 and its associated cytokine, IL-22 [57]. Upregulation of the anti-inflammatory cytokine IL-10 suggests its role in maintaining the cycle of negative feedback for chronic inflammation [57].

Genetic and autoinflammatory components of HS pathology have also been investigated, but more evidence is needed to reach definitive conclusions. Familial clustering, as well as mutations involving the γ-secretase complex, have been documented, but the complex genetics of HS remain a mystery [56,57]. Recent research also proposes the idea of classifying HS as an autoinflammatory disorder. Some suggest its classification as a neutrophilic dermatosis, a type of autoinflammatory condition, and others point to the autoinflammatory role of a dysregulated gamma-secretase/Notch pathway in disrupting the hair follicle’s protective root sheath and making it vulnerable to follicular occlusion [56,57]. Without a doubt, HS involves a variety of interconnected causes from genetic and environmental origin, but it all results in a series of complex molecular changes in the skin. Better understanding of the molecular mechanisms behind HS pathology is crucial for developing more effective treatments.

### 4.1. Innate Immunity Sensors (TLRs and NLRP Inflammasomes) in Hidradenitis Suppurativa 

The ongoing need for more HS treatment options has prompted the investigation of NLRP inflammasome inhibitors. NLRP3 inflammasome activity causes activation of the inflammatory cytokine IL-1β. IL-1β is highly implicated in the early stages of HS inflammation. Samples of HS lesions expressed increased levels of NLRP3, IL-1β, and the inflammasome-associated protease, caspase 1; elevated levels of all three molecules suggest overactivity of the NLRP3 inflammasome complex in HS lesions [58]. Another study of HS skin biopsy samples found that NLRP3 was not only overexpressed in lesional skin, but also in skin that appeared healthy and was non-lesional. Through comparing NLRP3 mRNA levels in lesional and non-lesional skin, it was found that NLRP3 mRNA was significantly higher in lesional skin [59]. The widespread upregulation of NLRP3 throughout HS patients’ skin, along with the uniquely high levels of NLRP3 mRNA in lesional skin, suggests the spread of inflammation from lesional to non-lesional skin through NLRP3 activity [59]. Additionally, a study assessing the in vitro addition of MCC950, an NLRP3 inflammasome inhibitor, resulted in a significant reduction in several inflammatory cytokines such as IL-1β, IL-17, and TNF-α in HS skin samples [60]. The NLRP3 inflammasome may be responsible for the positive feedback loop between IL-1β and IL-17 in HS pathology. The IL-17 released from Th17 stimulates NLRP3 activation, and stimulated NLRP3 increases the activity of IL-1β [61]. IL-1β then causes more inflammation and IL-17 activity by promoting the release of proinflammatory molecules such as antimicrobial peptides and proteins (AMPs) [60]. Blocking the activity of the NLRP3 inflammasome may prove particularly effective in decreasing coordination between proinflammatory cytokines and therefore reducing the ongoing propagation of chronic inflammatory signals in HS.

The IL-1R/TLR pathway plays a critical role in the pathogenesis of HS. Research has demonstrated that increased activation of this pathway is associated with elevated secretion of multiple downstream cytokines, including IL-1β, TNF-α, and IL-17, in both skin and blood samples from patients with HS [61,62]. TLRs and IL-1Rs share a common signaling mechanism through the Myddosome complex [63]. Upon ligand binding to TLRs or IL-1Rs, interleukin receptor associated kinase 4 (IRAK4) is recruited to the myeloid differentiation primary response 88 (MYD88) oligomeric complex [63]. This recruitment is pivotal as IRAK4 functions both as a scaffold and a kinase, facilitating downstream signaling through pathways such as NF-κB, mitogen activated protein kinases (MAPKs), and interferon regulatory factor 5/7 (IRF5/7) [61,62]. Given the central role of IRAK4 in these signaling pathways, understanding its expression and relationship to key inflammatory mediators in HS is essential. Such insights could provide a deeper mechanistic understanding of HS pathogenesis and help guide the development of more targeted therapeutic approaches.

### 4.2. Transcriptional Regulation (JAK/STAT and AHR Pathways) in Hidradenitis Suppurativa

Current research continues to focus on the potential use of JAK-STAT inhibitors for HS therapy. JAK-STAT pathways generate many of the pro-inflammatory cytokines involved in HS inflammation. STAT1 has been implicated in HS pathogenesis by inducing the transcription of TNF-α and IFN-γ [64]. Upregulation of JAK proteins may increase the production of pro-inflammatory cytokines in lesional skin. Recent clinical studies have revealed the clinical efficacy of using JAK1 inhibitors for HS therapy [65]. More research is needed to investigate the potential of inhibiting other JAK proteins such as JAK2, JAK3, and TYK2 [65].

Another target of ongoing HS research involves the transcription factor AHR. Stimulating AHR activity with the topical AHR agonist Tapinarof has shown clinical efficacy in treating other inflammatory skin diseases such as psoriasis and atopic dermatitis. Now, ongoing studies of another topical AHR agonist, AT193, are investigating its potential use in HS therapy [66]. Endogenous ligands such as tryptophan metabolites usually activate AHR [10]. One study indicated that disruption of tryptophan catabolism in HS-affected skin subsequently caused decreased activation of AHR [67]. As a transcription factor, AHR promotes the expression of several target genes such as AHRR, cytochrome P450 family 1 subfamily A member 1 (CYP1A1), and cytochrome P450 family 1 subfamily A member 2 (CYP1A2); expression of all three genes was significantly reduced in patients with HS, indicating reduced AHR function [10]. Decreased AHR in the skin may contribute to inflammatory processes in HS. Correcting the differential activity of AHR in HS may introduce the opportunity for novel treatment options.

### 4.3. Post-Transcriptional Regulation (miRNAs, lncRNAs, circRNAs) in Hidradenitis Suppurativa 

Dysregulation of miRNA in the skin lesions of HS patients may contribute to the pathologic mechanisms of the disease. Several proteins responsible for the proper functioning of miRNA in patients with HS, such as transactivation-responsive RNA-binding protein-1 (TRBP1) and metadherin, were shown to be dysfunctional in HS-affected skin [11]. Also, several types of miRNA are differentially expressed in patients with HS. For example, miR155-5p is overexpressed in HS-affected skin; it is thought to increase inflammation by stimulating the expression of proinflammatory cytokines such as IL-1β and TNF-alpha [68]. MiR-21-5p is also overexpressed in HS skin lesions. It activates T cells and increases expression of Th17-related cytokines. Several other miRNAs such as miR-146a-5p, miR-206, miR-338-3p, miR-24-1-5p, and miR26a-5p were all shown to be downregulated in HS lesions [68]. Differential expression of miR-146a-5p has been studied as a contributor to inflammation in several other diseases such as diabetes, cystic fibrosis, and CVD. Dysregulation of miR-146a-5p is thought to trigger inflammatory pathways by increasing IL-6 and TNF-α levels [68]. One overexpressed miRNA in particular, miR-338-5p, was identified as a potential biomarker for HS. Its abnormally high levels in HS lesions and its link to inflammatory cytokine production and disease invasiveness highlight its possible use as a non-invasive biomarker and target of treatment strategies in HS [68,69]. Diagnosis of HS proves particularly challenging, and many patients suffer for years without a diagnosis; using certain subtypes of miRNA as non-invasive biomarkers from serum samples may assist with early detection and more timely initiation of treatment.

LncRNAs play a vital role in normal physiological processes, including cell differentiation and tissue development. However, their aberrant expression is increasingly recognized in various pathological conditions. LncRNAs exert their regulatory functions by binding to specific effector molecules, either through sequence complementarity or structural recognition, thereby mediating gene expression [70]. During the differentiation of epidermal keratinocytes, transcriptional changes occur in which lncRNAs have been implicated in this differentiation process [71]. When deregulated, these lncRNAs contribute to aberrant keratinocyte differentiation and disrupt epidermal homeostasis [70]. This disruption is critically involved in the pathogenesis of several hyperproliferative skin diseases, including HS. Deregulated lncRNAs can upset the delicate balance between damaging and reparative processes in the skin, which exacerbates chronic inflammation, impairs wound healing, and hinders tissue renovation [71]. Although further research is needed to fully understand the role of lncRNAs in HS and to develop reliable biomarkers, the growing interest and investment in this area suggest that significant progress is being made.

The current literature reveals a significant gap in the understanding of the relationship between circRNA and HS. To date, there is an insufficiency of studies exploring this connection, leaving the potential roles of circRNAs in HS largely unexplored. However, as research in the field of non-coding RNAs and inflammatory skin disorders advances, circRNAs may emerge as crucial regulatory molecules in the pathogenesis and progression of HS.

### 4.4. Neuro-Immune Axis in Hidradenitis Suppurativa

The complex interplay between hormones and the pathogenesis of HS suggests a potential link to the neuro-immune axis, where hormonal fluctuations may influence immune responses and contribute to the chronic inflammation characteristic of the disease. The hormonal mechanisms underlying the pathogenesis of hidradenitis suppurativa remain incompletely understood, necessitating further research to clarify their contributions. Hormones, such as androgens and progesterone, are believed to be involved in the development and exacerbation of HS [72]. Clinical observations have highlighted patterns that suggest a hormonal influence on the disease. For instance, the onset and exacerbation of HS often coincide with puberty, a period marked by elevated levels of androgen and progesterone [73]. These hormones may contribute to the occlusion of hair follicles, a key factor in the development of HS lesions. The hypothesis of hyperandrogenism as a driving factor in HS is supported by several clinical features, including premenstrual flare-ups, the predominance of the disease in females, the occurrence of HS after menarche, and the reported improvement of symptoms during pregnancy [74]. Studies have documented that between 44% and 63% of women with HS experience exacerbations during the perimenstrual period, further underscoring the potential role of hormonal fluctuations in disease activity [74].

## 5. Systemic Sclerosis (Scleroderma)

The term scleroderma, although often used interchangeably with systemic sclerosis (SSc), specifically refers to the cutaneous manifestations of SSc. Scleroderma is classified as a chronic autoimmune skin disease. SSc, however, encompasses a broader spectrum of clinical features beyond the skin involvement of scleroderma; it includes internal organ involvement and diffuse fibroproliferative vascular modifications [75,76]. The clinical presentation and course of systemic sclerosis (SSc) are highly heterogeneous, with life expectancy mainly influenced by lung and heart involvement [75]. SSc predominantly affects women, with variations in disease severity and environmental exposure [75]. The initial presentation of scleroderma often involves Raynaud’s syndrome and hardening of the skin in the face and at the extremities such as the fingers. Ulcerations, necrosis, calcification, and even loss of fingertips may occur. Extensive telangiectasias and severe pruritus are also associated with scleroderma [76]. Pathogenesis involves altered homeostasis driven by genetic predisposition, environmental factors, and various triggers. Epigenetic modifications play a role in pathogenesis, leading to immune-inflammatory dysregulation, abnormal endothelial cell behavior, and myofibroblast trans-differentiation [75]. Inflammatory processes are thought to drive the initial response to a disrupted homeostasis; inflammation is also thought to cause the ultimate characteristic responses to homeostasis problems in SSc: fibrosis and structural vasculopathy [75].

The pathogenesis of inflammation in SSc involves type 2 helper (Th2) cells which release pro-fibrotic cytokines such as IL-4 and IL-13 [76]. The overabundance of the Th2 cytokines leads to increased production and differentiation of fibroblasts and increased collagen synthesis [76]. Cytokines IL-4 and IL-13 cause macrophages and fibroblasts to release high levels of TGF-β. TGF-β is a key cytokine in many pro-inflammatory and pro-fibrotic mechanisms in SSc [76]. IL-4 and IL-13 also trigger the activation of B cells, as well as the production of immunoglobulin and adhesion molecules such as intracellular adhesion molecule 1 (ICAM1), which facilitates the invasion of more Th2 cells into areas with increased TGF-β levels [76]. Mast cells also accumulate in fibrotic lesions and, apart from releasing histamine, they secrete fibrogenic mediators themselves [76]. Other T cell subtypes such as Th17 are also thought to play a role in the inflammatory processes of SSc, but the exact mechanisms are still under investigation [76].

### 5.1. Innate Immunity Sensors (TLRs and NLRP Inflammasomes) in Systemic Sclerosis

Toll-like receptors (TLRs) play a pivotal role in recognizing pathogens and internal activation signals, leading to inflammation and alterations in innate immunity associated with SSc. The interaction between DAMPs and TLRs on fibroblasts directly activates these cells to produce large amounts of collagen, contributing to ECM expansion typical of SSc [77]. The inflammatory actions of TLR4, in particular, have been implicated in the development of fibrosis through sustained activation of fibroblasts. Inhibiting TLR4s may emerge as a novel therapy for SSc [77].

The NLRP3 inflammasome may contribute to the development and expansion of SSc. The expression of NLRP3 and its downstream proteins, including caspase-1, IL-1β, and IL-18, are increased in the serum and skin biopsies of SSc patients. The NLRP3/IL-1β signaling pathway may play a role in regulating the T and B cells involved in SSc inflammation [78]. Also, the expression of molecules involved in the NLRP3 inflammasome complex, such as IL-1β and caspase-1, is increased in SSc patients with vascular damage compared to those without. Therefore, the NLRP3 inflammasome may contribute to vasculopathy [78]. Additionally, more severe levels of skin fibrosis in SSc patients correlate with a higher expression of NLRP3 inflammasome components. Targeting the NLRP3 inflammasome for SSc therapy has yet to be sufficiently explored, but promising results from using NLRP3 inhibition in cases of pulmonary fibrosis—well as fibrosis in the liver, myocardium and kidney—indicate the potential of using similar inhibitors to reduce the significant fibrosis associated with SSc [78].

### 5.2. Transcriptional Regulation (JAK/STAT and AHR Pathways) in Systemic Sclerosis 

Signaling pathways involving the AHR transcription factor seem to influence the progression of SSc. SSc is a complex disease characterized by aberrant signaling of TGF-ß, which plays a critical role in the molecular mechanisms of the disease. The extracellular matrix (ECM) protein degradation is tightly controlled by matrix metalloproteinases (MMPs) and their inhibitors, tissue inhibitors of metalloproteinases (TIMPs) [79]. One significant molecule in this context is 6-formylindolo[3,2-b]carbazole (FICZ), a tryptophan photo-product. FICZ binds with high affinity to the AHR [80]. Upon binding, activated AHR translocates from the cytoplasm to the nucleus, leading to the transcription of target genes, such as CYP1A1 and CYP1B1, in human keratinocytes and fibroblasts, respectively [80]. The AHR signaling pathway is crucial for UV (ultraviolet) light response regulation, as UV exposure upregulates CYP1A1 and CYP1B1 expression [81]. This upregulation is negated by AHR deficiency or the presence of a selective AHR antagonist. FICZ notably upregulates MMP1 expression through the activation of the MEK/ERK signaling pathway within the MAPK cascade, whose actions are dependent on the AHR signaling pathway [80]. Due to its potent inhibitory effect on collagen synthesis, the AHR ligand FICZ shows potential as a therapeutic agent for treating fibrosing or sclerotic diseases when applied exogenously.

Recent literature has highlighted the significant activation of the JAK/STAT pathway in SSc biopsies and disease models [82,83]. Analysis of SSc transcriptomes revealed that skin biopsies have exhibited significantly elevated levels of a consensus IL-6/JAK/STAT3 signature based on experimentally derived gene expression [82]. Furthermore, immunohistochemistry has similarly demonstrated the presence of activated JAKs and STAT3 in both SSc skin and lung biopsies [82,83]. These combined transcriptome and protein data sets suggest that the disease process in specific subsets of SSc patients is directly driven by the activation of the JAK/STAT3 pathway [83]. Consequently, these patients might respond favorably to therapeutic interventions targeting this pathway, potentially leading to the normalization of aberrant gene signatures and the resolution of tissue fibrosis.

### 5.3. Post-Transcriptional Regulation (miRNAs, lncRNAs, circRNAs) in Systemic Sclerosis

Based on current research, approximately 40 microRNAs have been implicated in fibrotic diseases [84]. Many of these microRNAs modulate fibrosis by targeting connective tissue growth factor (CTGF), extracellular matrix proteins, the TGF-β signaling pathway, and the mitogen-activated protein kinase (MAPK) pathway [85]. Additionally, certain microRNAs influence fibrogenesis by modulating epithelial-to-mesenchymal transition (EMT) or by stimulating the proliferation of myofibroblasts. Specifically, in SSc, miR-138 and miR-27a inhibit key pathways involved in EMT and subsequent fibrosis [85]. The expression levels of miR-138 and miR-27a are significantly reduced in patients with SSc compared to healthy controls, with miR-138 being further decreased in diffuse cutaneous SSc [85]. This suggests that both miRNAs could serve as diagnostic biomarkers, with miR-138 potentially indicating disease severity. Among the miRNAs significantly upregulated in SSc, miRNA-21-5p has been extensively studied for its role in skin fibrosis. Elevated miRNA-21-5p levels are observed in the serum of SSc patients, and its expression is increased by TGF-β stimulation in skin fibroblasts [86].

The biological significance of four lncRNAs—ANCR, TINCR, HOXA distal transcript antisense RNA (HOTTIP), and SPRY4-intronic transcript 1 (SPRY4-IT1)—has been established based on their known roles in skin biology [87]. Each of these lncRNAs is mechanistically linked to various physiological and pathophysiological processes in the skin, including wound healing, inflammation, and fibrosis. Three of these lncRNAs—SPRY4-IT1, HOTTIP, and ANCR—show a correlation with the modified Rodnan skin score (MRSS), which measures skin thickness and is often used as a marker of disease severity in SSc [87]. Among them, SPRY4-IT1 is particularly notable for its correlation with specific disease phenotypes. Moreover, SPRY4-IT1 demonstrated superior diagnostic accuracy compared to the other studied lncRNAs, with its upregulation serving as a predictor of SSc risk [87]. This suggests that SPRY4-IT1 could serve as a valuable surrogate biomarker for the diagnosis of SSc. In addition, the studies have highlighted a pattern of plasma ANCR downregulation and TINCR upregulation in SSc patients [87]. This imbalance between ANCR and TINCR aligns with their roles in keratinocyte differentiation, which may lead to increased differentiation of keratinocytes in the skin epidermal layer [87]. Consequently, this results in a thickened epidermis and hypertrophic keratinocytes, further contributing to the pathology observed in SSc.

The current body of research on SSc has yet to thoroughly investigate the potential role of circRNA in the disease’s progression. At present, there is a noticeable lack of studies examining the relationship between circRNAs and SSc, which leaves a gap in our understanding of how these non-coding RNAs might influence the complex molecular mechanisms underlying this autoimmune disorder and other fibrotic diseases.

### 5.4. Neuro-Immune Axis in Systemic Sclerosis

Since the early studies conducted on SSc, the autonomic nervous system (ANS) has been recognized as playing a concurrent pathogenic role in SSc pathogenesis. These studies initially identified the presence of autonomic neuropathy in SSc patients, suggesting a link between ANS dysfunction and the disease’s pathogenesis [88]. One method used to assess autonomic function is the sympathetic skin response (SSR), a non-invasive technique that measures changes in skin conductance [89]. These changes occur due to the activation of sweat glands, which are under the neural control of sympathetic cholinergic sudomotor fibers. SSR provides valuable insight into the functioning of the sympathetic nervous system in SSc patients [89]. Research findings have consistently shown that SSR is either absent or significantly delayed in the skin of individuals with SSc [89]. These abnormalities in SSR reflect the broader autonomic dysfunction associated with the disease and highlight the role of the ANS in the pathophysiology of SSc.

## 6. Discussion and Future Directions

In order to hijack the ruthless destruction of skin diseases, we must dig deeper and unveil the complex molecular mechanisms which establish the framework of their pathology. Only by analyzing skin diseases at a molecular level can we later develop a big picture understanding of their clinical implications. The study of molecular pathology also reveals numerous new avenues for targeted treatment strategies in skin disease therapies. Many patients suffering from inflammatory conditions such as psoriasis, atopic dermatitis, hidradenitis suppurativa, and scleroderma lack the therapeutic resources to effectively manage their disease. Researchers are constantly searching for new ways to disrupt the pathologic processes of skin diseases and decrease inflammation. The recent emergence and growing popularity of medications such as biologics in dermatological therapies, which work on a molecular level to block components of pathologic mechanisms, emphasize the importance of a detailed appreciation for submicroscopic processes in the skin. Ongoing research of inflammatory molecular mechanisms such as the actions of innate immunity sensors, the regulation of transcription, the epigenetic influences of non-coding mRNAs, and neural-immune interactions has highlighted endless opportunities for unlocking the mysteries of skin disease.

Looking forward, more research regarding the inflammatory mechanisms of skin diseases must be conducted. Significant gaps exist, especially regarding the pathogenic role of lncRNAs and circRNAs. A more thorough understanding of the neuro-immune axis could prove revolutionary in providing treatment for patients with skin disease. More attention must be given to analyzing the complex interactions between the nervous and immune systems. While all mechanisms discussed reveal potential for the development of therapeutic advancement, more targeted research must be conducted to further validify the development of new biological drugs and the use of inflammatory molecules as biomarkers of disease. Exciting new discoveries within the discipline of molecular pathology have furthered our understanding of inflammatory processes in skin disease. The emerging success of applying such knowledge to therapeutic advancements inspires a continued focus on analyzing molecular processes for the sake of providing relief and hope for the millions of people affected by skin disease worldwide.
